# Supplementary material for: Nobiletin Prevents D-Galactose-Induced C2C12 Cell Aging by Improving Mitochondrial Function
Source: Int J Mol Sci. 2022 Oct 8;23(19):11963. doi: 10.3390/ijms231911963 (PMC9569543; doi:10.3390/ijms231911963)
Supplement: Supplementary file 1 [file ijms-23-11963-s001.zip › Supplementary Figure S1.pdf]

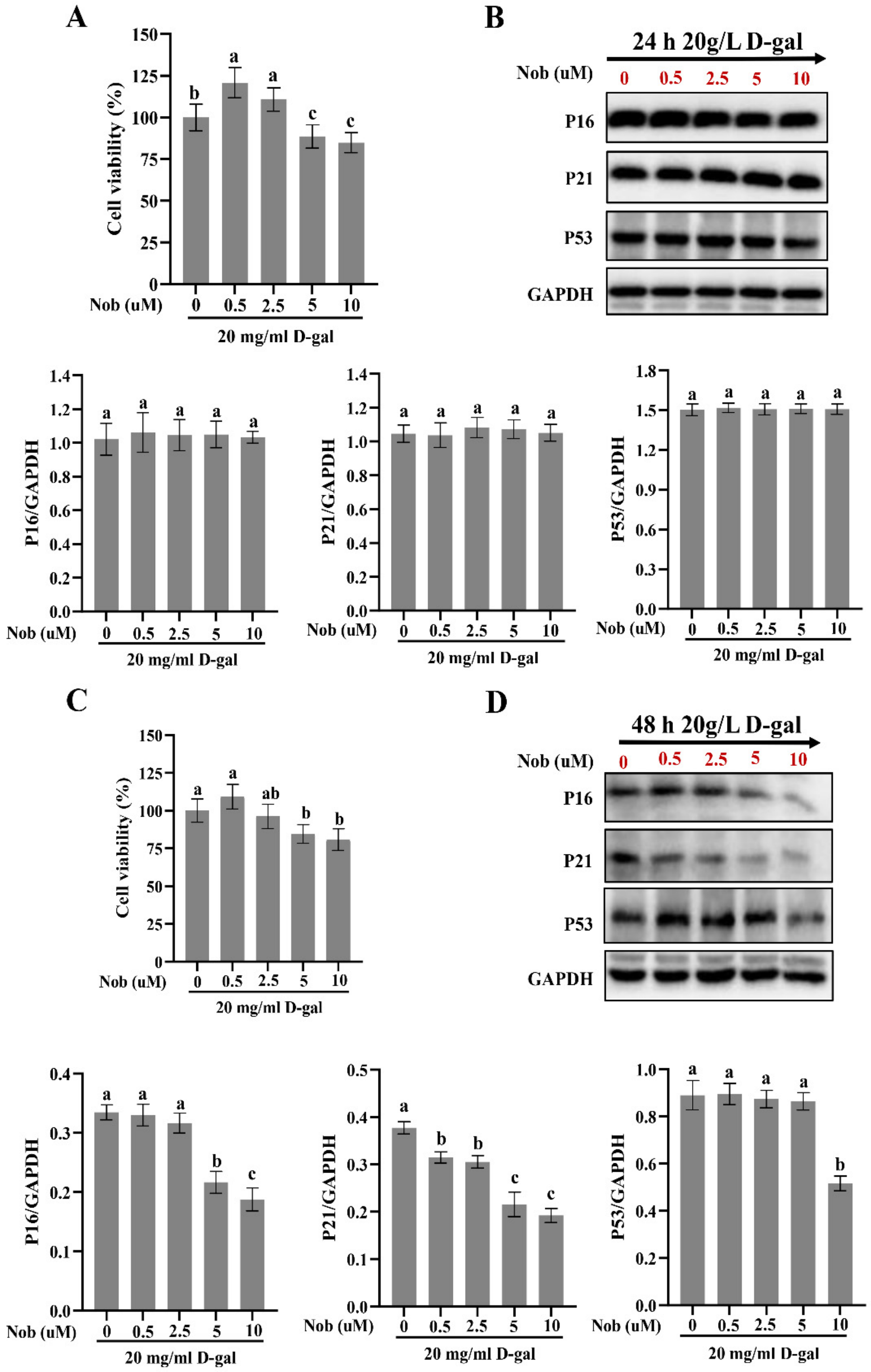

Supplementary Figure S1. Effects of different concentrations of Nob on cell viability and senescence markers in D-gal-induced senescent cells. (A) Cell viability in D-gal-induced C2C12 myoblasts treated for 24 h with different concentrations of Nob. (B) Western blot analysis of P16, P53, P21, and GAPDH in D-gal-induced C2C12 myoblasts treated for 24 h with different concentrations of Nob. (C) Cell viability in D-gal-induced C2C12 myoblasts treated for 48 h with different concentrations of Nob. (D) Western blot analysis of P16, P53, P21, and GAPDH in D-gal-induced C2C12 myoblasts treated for 48 h with different concentrations of Nob. Different lowercase letters represent significant differences between among different treatment groups ($P<0.05$).
